# Characterization of the Luminal and Mucosa-Associated Microbiome along the Gastrointestinal Tract: Results from Surgically Treated Preterm Infants and a Murine Model

**DOI:** 10.3390/nu13031030

**Published:** 2021-03-23

**Authors:** Ingeborg Klymiuk, Georg Singer, Christoph Castellani, Slave Trajanoski, Beate Obermüller, Holger Till

**Affiliations:** 1 Center for Medical Research, Medical University of Graz, 8010 Graz, Austria; ingeborg.klymiuk@medunigraz.at (I.K.); slave.trajanoski@medunigraz.at (S.T.); 2 Department of Paediatric and Adolescent Surgery, Medical University of Graz, 8010 Graz, Austria; christoph.castellani@medunigraz.at (C.C.); beate.obermueller@medunigraz.at (B.O.); holger.till@medunigraz.at (H.T.)

**Keywords:** intestinal microbiome, mucosa, stool, neonates, mice

## Abstract

Environmental factors, including nutritional habits or birth mode, are known key determinants for intestinal microbial composition. Investigations of the intestinal microbiome in different species in a multiplicity of studies during recent decades have revealed differential microbial patterns and quantities along the gastrointestinal (GI) tract. Characterization of the microbial pattern in various aspects is a prerequisite for nutritional interventions. In this 16S rRNA amplicon-based approach, we present a characterization of the mucosa-associated microbiome in comparison with the luminal community of four infants at the time of the closure of ileostomies and perform a systematic characterization of the corresponding luminal and mucosal microbiome from jejunal, ileal and colonic regions, as well as collected feces in mice. The most dominant taxa in infant-derived samples altered due to individual differences, and in the mucosa, *Enterococcus*, *Clostridium* *sensu* *stricto* *1*, *Veillonella*, *Streptococcus* and *Staphylococcus* were the most abundant. Two less abundant taxa differed significantly between the mucosa and lumen. In murine samples, relative abundances differed significantly, mainly between the intestinal regions. Significant differences between mouse mucosa- and lumen-derived samples could be found in the observed species with a trend to lower estimated diversity in mucosa-derived samples, as well as in the relative abundance of individual taxa. In this study, we examined the difference between the mucosal and luminal bacterial colonization of the gastrointestinal tract in a small sample cohort of preterm infants. Individual differences were characterized and statistical significance was reached in two taxa (*Cupriavidus*, *Ralstonia*). The corresponding study on the different murine intestinal regions along the GI tract showed differences all over the intestinal region.

## 1. Introduction

Facilitated by the rapid developments in molecular biology techniques, our understanding of the human microbiome, the naturally occurring communities of microbes, has increased dramatically during recent decades [1]. Today, we know that the healthy, adult human body is colonized by a vast number of microbes, even in habitats thought to be sterile for a long time [2]. In infants, the main colonialization with microbes starts immediately after birth [3,4], and research on the dynamics and development of an emerging human microbiome is of superior interest to understand the human microbial community and subsequently use these findings for the development of future therapeutic strategies [5]. The early microbiome represents a very fragile and vulnerable community susceptible to negative perturbations [6] as well as for positive interventions by the application of, for example, pre- and probiotics [7,8,9], to develop a stable adult gut community [10].

One of the key questions that still need to be answered is “what is normal” during the shaping of the human microbial community. Therefore, accurate characterization and understanding of the infant gut microbiome during the first years of life and over the different intestinal regions is a fundamental prerequisite for all therapeutic aspects. Under physiological conditions, the healthy newborn microbiome during the first days of life is dominated by Actinobacteria (mainly *Bifidobacterium*), Firmicutes, Proteobacteria (with *Lactobacillus* spp. resembling the vaginal microbiome) and Bacteroidetes [11,12]. The newborn microbiome, however, is significantly affected by the mode of delivery [13] and nutritional habits such as breast or formula feeding [14] as well as environmental factors such as frequent disinfection or the application of antibiotics [15]. Breast milk oligosaccharides, fatty acids and other milk components have been described to promote the abundance of *Bifidobacterium* and other advantageous taxa and prevent pathogenic bacteria to bind to host cell receptors [16,17]. The introduction of plant oligosaccharides and solid food later during weaning causes a shift of the infant’s microbiome in the direction of the phyla Bacteriodetes (*Bacteroides*) and Firmicutes (*Clostridium*, *Streptococcus*) [18] and also reduces the abundance of Actinobacteria (*Bifidobacterium*) [12]. Finally, the interaction between the host immune system and the microbiota is known to shape the intestinal community during development as an essential factor for the training and development of the host’s innate and adaptive immune system [19,20].

The gut mucosa plays a unique role as an interaction space between the host’s immune system and the microbial community. Mucosa- and lumen-associated microbiota differ in their pattern, and nutritional supplementation might have a different impact on the respective microbial communities. In particular, the mucosa-associated microbiome plays a pivotal role in the development of the innate immune system in infants and in the long-term defense against bacterial pathogens such as *Clostridium* sp. or *Salmonella* sp. [21]. From studies of the entire gastrointestinal (GI) tract of adult patients, we know that the mucosa of the intestine is dominated by Bacteroidetes, Firmicutes and Proteobacteria, with distinct profiles along the different sections [22]. For example, Proteobacteria (*Pseudomonadaceae*) and Firmicutes (*Veillonellaceae*, *Streptococcaceae*) are reported to dominate the mucosa of the upper GI tract of adults but decrease in favor of Bacteroidetes (*Bacteroidaceae*, *Rikenellaceaea*, *Bacteroides and Prevotella*) in the lower GI tract [22,23]. A systematic analysis of the mucosa-associated landscape of the adult, healthy GI tract confirms the varying microbial profiles along the GI tract shaped by different niche factors along the intestine such as oxygen level, nutrients, ph and others [23].

However, data on the mucosa-associated microbiome of human neonates and infants and potential differences to the luminal microbiome are rare and valuable as this early time is of decisive meaning for the later mature microbiome. Studies performed in neonatal piglets have shed light on the development of the intestinal and mucosa-associated microbiome during the first days of life and the codevelopment of the immune system. The mucosa of the small and large intestine shows different dynamics concerning postnatal bacterial colonization related to divergent functions of the different intestinal regions [24]. Although a multiplicity of studies has analyzed the fecal microbiome of neonates and infants, the influence of administration of pre- and probiotics or the effect of formula feeding and nutritional supplementation [25,26,27,28], little is known about the differences of the infants’ microbiome along the GI tract and the particularities of mucosa and stool. It is precisely this information that is of great importance in order to develop therapeutic interventions in severe pediatric diseases, including necrotizing enterocolitis [29].

The aim of this study was therefore to characterize the luminal versus the mucosal bacterial microbiome in surgically treated preterm infants after enterostomy (ileostomy) creation. We show the bacterial colonization in both tissue types, and we thus provide data that represent an important basis for evaluating microbial development in early childhood. Further, we hypothesize to find corresponding differences in mucosa- and lumen-associated microbial colonization in a mouse model over the different intestinal regions. Therefore, we show the bacterial composition in mice by comparing the lumen and mucosa, and we analyze the course of the microbial composition along the GI tract in mice as a model organism. We sought to describe differences (1) between the mucosa-associated (resident) and luminal (transient) microbiome in the gastrointestinal tract in surgically treated pediatric patients the first time and (2) along the gastrointestinal tract in mice, demonstrating the overriding message of our work on preterms.

## 2. Materials and Methods

### 2.1. Human Measurements

Following ethical approval (EK 28-362 ex15/16) and informed written consent of the parents or legal guardians, the luminal and mucosal intestinal microbiome was assessed in four infants treated with ileostomies at the time of closure of the stomata. Before the operation, stool was collected from the proximal stoma and immediately stored at −80 °C. During the operation, an annular segment of the stoma was removed, carefully washed with phosphate-buffered saline (PBS) and stored at −80 °C. Detailed clinical information of the four included infants is shown in Table 1.

### 2.2. Murine Measurements

Fecal samples of eight nonpregnant female mice (B6CBAF2) were collected prior to euthanasia. Thereafter, mice were euthanized, the gastrointestinal tract was removed and the jejunum (3 cm distally of the ligament of Treitz), ileum (3 cm proximally of the ileocecal valve) and colon (3 cm distally of the ileocecal valve) were separated and opened on the antimesenterial side. The contents from each segment were collected to assess the luminal microbiome. Any residual contents were removed by thoroughly rinsing the bowel segments with PBS. Samples of 2 × 2 mm were collected for analysis of the mucosal microbiome. All samples were stored at −80 °C until further processing.

### 2.3. Total DNA Isolation, Library Preparation and Sequencing

Total DNA isolation was performed as published in Klymiuk et al. [30] with the MagNA Pure LC DNA III Isolation Kit (Bacteria, Fungi) (Roche, Mannheim, Germany) in a MagNA Pure LC device (Roche, Mannheim, Germany) with some modifications. Briefly, biopsy samples were homogenized in 500 µL of bacterial lysis buffer in MagNA Lyser Green Bead tubes (Roche, Mannheim, Germany) at 6500 rpm for 30 s three times. Samples were treated with 25 µL of Lysozyme (100 mg/mL) at 37 °C for 30 min and 43.4 µL of Proteinase K (43.4 µL at 20 mg/mL) at 65 °C overnight. After heat inactivation at 95 °C for 10 min, 250 µL of the sample was used in the MagNA Pure according to the manufacturer’s instructions, and total DNA was eluted in 100 µL of elution buffer. For stool or luminal samples, 75 mg sample was mixed thoroughly with 500 µL PBS. A 250 µL volume of the stool suspension was mixed with 250 µL of bacterial lysis buffer in MagNA Lyser Green Bead tubes (Roche, Mannheim, Germany) and mechanically treated at 6500 rpm for 30 s two times. Samples were treated with Lysozyme and Proteinase K (for 1 h) as described for the mucosa samples and loaded to and processed at the MagNA Pure LC device according to the manufacturer’s instructions. Total DNA was eluted in 100 µL as described above. DNA was stored at −20 °C until analysis. For PCR 16S rRNA amplification, 5 µL of biopsy-derived DNA and 2 µL of stool-derived DNA were used in a 30-cycle PCR reaction with the primers 27F-AGAGTTTGATCCTGGCTCAG and 357R-CTGCTGCCTYCCGTA spanning the V1-2 hypervariable regions [31], as published before [30]. Triplicates were pooled, 7.5 µL of the pool were used in an 8-cycle indexing PCR and 5 µL of each sample pooled to the final library. A 30 µL volume of the library was loaded to a 1.5% agarose gel and purified using the QIAquick Gel Extraction Kit (Qiagen, Hiden, Germany) according to the manufacturer’s instructions. After QC on an Agilent 2100 BioAnalyzer, samples were sequenced on an Illumina MiSeq desktop sequencer (Eindhoven, The Netherlands) with v3 chemistry for 2 × 300 cycles. FastQ files were used for data analysis.

### 2.4. 16S Data Analysis

A total of 976,241 (human) and 8,585,778 (mouse) MiSeq paired-end raw sequence reads were quality filtered, denoised, dereplicated, merged and checked for chimeras using the DADA2 pipeline [32] with standard settings as implemented in the QIIME2 2018.4 microbiome bioinformatics platform [33] integrated into an own nonpublic instance of Galaxy [34]. Taxonomic assignment of the DADA2 [32] representative sequences was provided with the QIIME2 sklearn-based classifier against the SILVA rRNA database Release 132 at 99% identity [35]. A phylogenetic tree was created with FastTree on Mafft-aligned representative sequences [36]. Further downstream statistical data analysis, including alpha and beta diversity, was conducted with the R 3.5.3 program for statistical computing. Summarized absolute counts from an OTU table on genus level were used to assess the abundant changes of each genus between the luminal and tissue samples. To calculate the size factors for the normalization of the feature table, we used the GMPR method, which was specially developed for microbiome (zero-inflated count) data [37]. Significant differential abundant changes between investigated groups were tested using the DESeq2 method [38] that uses shrinkage estimation for dispersions and fold changes by fitting a generalized linear model to each taxa. To correct for type I errors from multiple comparisons, the Benjamini–Hochberg approach was applied.

## 3. Results

On average, 122,030 raw sequence reads were analyzed per human sample (range: 11,092 to 232,757) and 153,317 per mouse-derived sample (range: 12,976 to 263,761). Rarefaction levels were set to 5800 in human-derived samples and to 9200 in murine samples. All FastQ raw data can be accessed through the SRA accession number PRJEB42814 at the European Nucleotide Archive (ENA).

### 3.1. Human-Derived Samples: Bacterial Pattern in the Mucosa and Corresponding Luminal Samples

**Figure 1:**
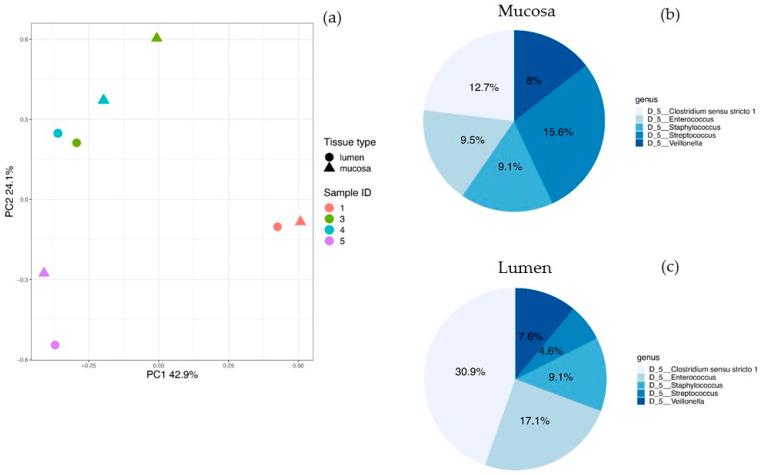
PCoA clustering and the most abundant genera in human samples. (**a**) PCoA plot with weighted UniFrac analysis of human luminal and mucosa samples revealed clustering according to the proband rather than the tissue type. Dot colors indicate the different probands, circles are lumen-derived samples and triangles are mucosa-derived samples. Pie chart of the five most abundant genera of the (**b**) mucosal and (**c**) luminal bacterial microbiome.

**Figure 2:**
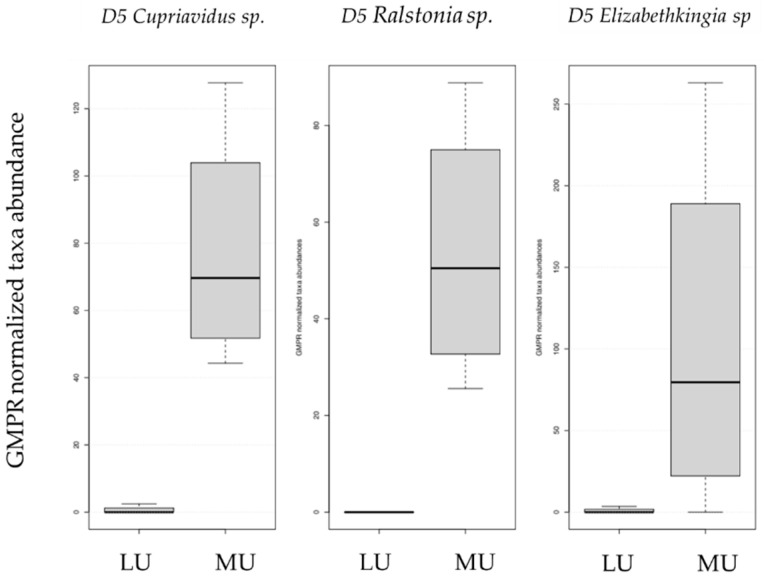
Genus-level significance plots of two genera significantly different in their abundance between mucosa- and lumen-derived samples in human infant samples and one genus that just missed the statistical significance. *Cupriavidus* (adj. *p*-value = 0.029); *Ralstonia* (adj. *p*-value = 0.029). *Elizabethkingia* (adj. *p*-value = 0.078) just missed statistical significance. For relative abundances and absolute read numbers, see Supplementary Table S2. LU: lumen, MU: mucosa.

For this study, four children (aged two to nine months) with ileostomies due to various reasons were included (Table 1). Differences in the bacterial pattern and abundance between the mucosa and lumen were analyzed in these pediatric cohort samples. Alpha diversity metrics analysis (phylogenetic diversity (PD) whole tree) revealed significantly higher phylogenetic diversity levels in mucosa-derived samples compared to luminal ones (*p*-value = 0.031). Other performed alpha diversity calculations (observed operational taxonomic units, OTUs, *p*-value = 1.00, Shannon diversity index *p*-value = 0.625, PD Faith = 0.125) did not result in statistically significant differences between luminal and mucosal samples. Beta diversity calculations did not result in statistically significant differences between the two sample groups (Anosim Bray–Curtis *p*-value = 0.725, Anosim weighted UniFrac *p*-value = 0.646, Adonis Bray–Curtis *p*-value = 0.827, Adonis weighted UniFrac *p*-value = 0.659). Weighted UniFrac-based PCoA clustering exhibited clustering according to the proband rather than the tissue type (Figure 1a). Furthermore, the five most abundant genera found in the human mucosa samples were *Streptococcus*, *Clostridium sensu stricto 1*, *Enterococcus*, *Staphylococcus* and *Veillonella* (Figure 1b and Supplementary Table S1). The most abundant phyla in lumen and mucosa samples were Firmicutes (mucosa: 77.24%, lumen: 85.63%) followed by Proteobacteria (mucosa 14.97%, lumen 5.52%) and Bacteroidetes (mucosa 4.62%, lumen 6.83%) but without statistically significant differences between the mucosa and lumen (adj. *p*-value Firmicutes = 0.804, *p*-value Proteobacteria = 0.675, *p*-value Bacteroidetes = 0.984). DSeq2 analysis at the genus level, however, revealed significant differences for *Cupriavidus* (adj. *p*-value = 0.029; 435 reads and 0.52% relative abundance in the mucosa) and *Ralstonia* (adj. *p*-value = 0.029; 314 reads and 0.38% relative abundance in the mucosa) with an increased abundance of all three taxa in the mucosa compared to luminal samples (Figure 2 and Supplementary Table S2). *Elizabethkingia* (adj. *p*-value = 0.078; 1120 reads and 1.35% relative abundance in the mucosa) just missed the statistical significance but should still be considered here as the most abundant from those three. However, there were no significant differences between the specimen types over all infants. In luminal samples of the probands, the five genera were found with different frequencies, and the most abundant genera were *Clostridium sensu stricto 1*, *Enterococcus*, *Staphylococcus*, *Veillonella* and *Streptococcus* (Figure 1c and Supplementary Table S2).

### 3.2. Mouse-Derived Samples: Differences in Tissue Type and Characterization along Intestinal Passages

**Figure 3:**
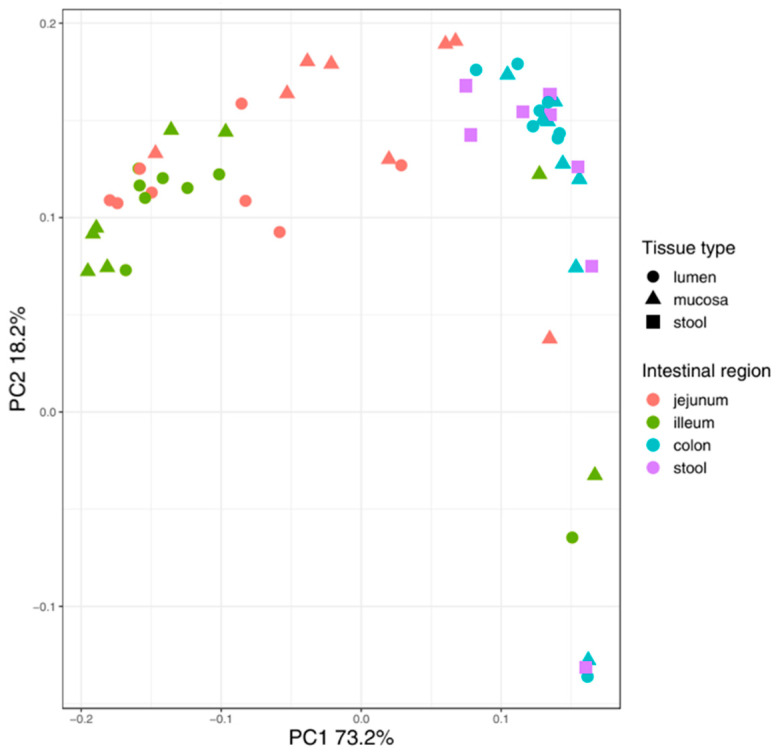
PCoA plot of weighted UniFrac analysis of mouse luminal and mucosa samples. Stool samples were collected from the anus. Clustering along the intestinal regions rather than the tissue type was observed, except for jejunal samples, where mucosa- and lumen-derived samples differed significantly (adj. *p*-value = 0.045).

To compare and interpret the rare and extremely valuable data derived from infant samples, we additionally analyzed the differences of the bacterial pattern between mucosal and luminal bacterial DNA in mice along the three intestinal regions (jejunum, ileum and colon) and finally in comparison to the freshly sampled fecal pellet. Alpha diversity calculations revealed significant differences in the observed species (adj. *p*-value = 0.047) but not in the Shannon diversity index (adj. *p*-value = 0.445) between the mucosa and lumen in the jejunum (Table 2). All other intestinal regions analyzed did not reveal statistically significant differences in alpha diversity calculations between the lumen and mucosa (Table 2). Adonis testing on weighted UniFrac distance values revealed statistically significant differences between the mucosa and lumen in the jejunum only but not in the other investigated intestinal regions (Table 2). PCoA clustering of weighted UniFrac distances showed clustering of samples according to the intestinal region rather than the sample origin (lumen/mucosa) with the exception of the jejunal samples (Figure 3). Correspondingly, DESeq-based analysis of differential abundances between the mucosa and lumen in jejunal samples calculated over all mice revealed *Lachnospiraceae NK4A136 group *sp., *Romboutsia* sp., *Turicibacter *sp., *Escherichia-Shigella *sp. and *Akkermansia *sp. to differ significantly (Supplementary Table S3). Nevertheless, the relative abundances of the ileal samples of the genera *Romboutsia *sp., *Lachnospiraceae* ssp., *Lachnospiraceae NK4A136 group* as in the jejunal tissue, *Escherichia-Shigella *sp., *Akkermansia *sp., *Enterococcus *sp. and four more taxa beyond a baseMean value of 100 (Supplementary Table S3) were significantly different in mucosa compared to lumen samples. Additionally, *Lachnospiraceae other *sp. and *Roseburia *sp. were taxa differentially abundant in colonic samples (Supplementary Table S3).

Shannon diversity indices did not differ between these sample groups in mucosa-derived specimens at all. The Shannon diversity indices in lumen-derived samples along the GI tract showed significance between jejunum vs. colon (adj. *p*-value = 0.047) and ileum vs. colon (adj. *p*-value = 0.047) but not between jejunum vs. ileum and colon vs. feces, respectively (Table 2). Observed species did not reveal statistically significant results in any of the described comparisons. In lumen-derived samples along the GI tract, we found significant beta diversity differences (Adonis, weighted UniFrac) in jejunum vs. colon (adj. *p*-value = 0.004) and ileum vs. colon (adj. *p*-value = 0.004) but not in the comparisons of jejunum vs. ileum or colon vs. feces (Table 2). Analyzing the different intestinal regions, we found beta diversity differences of mucosal samples (Adonis, weighted UniFrac) in the comparison of jejunum vs. colon (adj. *p*-value = 0.004) and ileum vs. colon (adj. *p*-value = 0.004) but no significant differences comparing jejunum and ileum (adj. *p*-value = 0.200) and neither in colon vs. feces (adj. *p*-value = 0.324) (Table 2).

**Figure 4:**
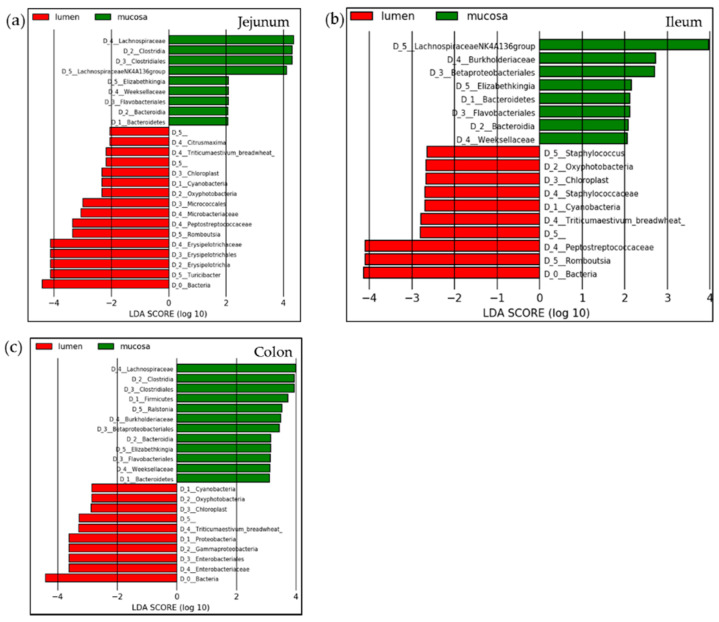
Linear discriminant (LDA) and effect size (LefSe) analysis for mucosa versus luminal samples along the GI tract: (**a**) jejunum, (**b**) ileum and (**c**) colon in mice.

As differential abundant genera from DESeq analysis in the mouse mucosa along the examined intestinal regions, we identified, with a baseMean over 100, *Romboutsia *sp. in the comparison of jejunum vs. ileum, *Turicibacter *sp., *Lachnospiraceae NK4A136 group *sp., an uncultured *Lachnospiraceae* genus, *Romboutsia *sp., two *Enterobacteriaceae* genera and *Lachnoclostridium *sp. in the comparison of ileum vs. colon and two *Enterobacteriaceae* genera and *Lachnoclostridium *sp. in the comparison of jejunum vs. colon (Supplementary Table S4). In the luminal samples, we found the differentially abundant genera *Turicibacter *sp., *Lachnospiraceae NK4A136 group *sp., an unclassified *Lachnospiraceae* genus, *Romboutsia *sp., *Enterobacteriaceae*, *Enterobacter *sp., *Lachnoclostridium *sp., *Enterococcus *sp. and *Sporosarcina *sp. in the comparison of ileum vs. colon (Supplementary Table S5). Analyzing jejunum vs. colon we found similar taxa to be differently abundant namely *Turicibacter *sp., *Lachnospiraceae NK4A136 group*, *Lachnospiraceae*, *Romboutsia *sp., *Enterobacteriaceae*, *Enterobacter *sp., *Lachnoclostridium *sp., *Clostridium sensu stricto 1 *sp. and *Sporosarcina *sp. (Supplementary Table S5). Moreover, the comparison of jejunum vs. ileum in the luminal samples resulted only in three relevant taxa with significant differences in abundances, namely *Romboutsia *sp., *Enterobacteriaceae* and *Lachnoclostridium *sp. (Supplementary Table S5). Additionally, for differential abundant taxa analysis, we performed LefSe calculations on lumen vs. mucosa in jejunum, ileum and colon samples (Figure 4a–c). On the D5 level in jejunum samples *Turicibacter*, *Romboutsia* and *Elizabethkingia* were significantly differentially abundant genera between the lumen and mucosa, although *Elizabethkingia* had a relative abundance of less than 1% (Figure 4a). In ileum-derived samples, the genera *Romboutsia* and *Lachnospiraceae NK4A136 group* were significantly different, with a relative abundance above 1% (Figure 4b), and in colonic samples, none of the significantly different genera had a relative abundance of more than 1%. The significant differences in this intestinal region were found at the family or order level (Figure 4c and Supplementary Table S6).

**Figure 5:**
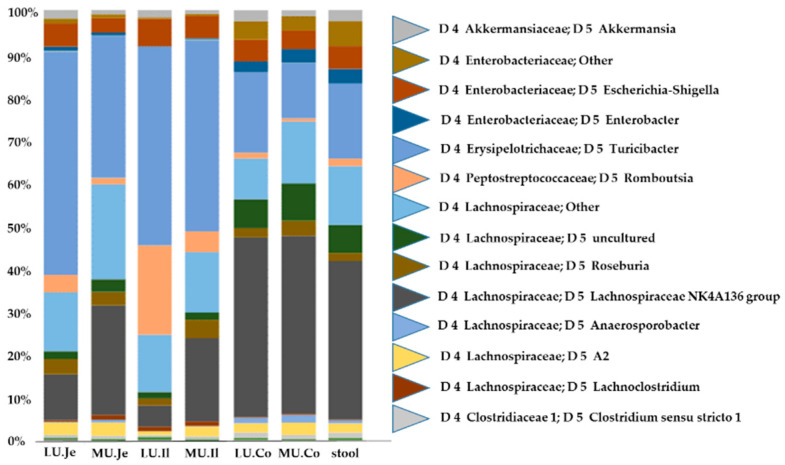
Bar charts of relative abundances of bacterial genera in the lumen (LU) and mucosa (MU) along the murine gastrointestinal tract (jejunum, ileum, colon, feces) over all mice studied. The 29 most abundant genera are presented (14 most dominant named individually), with at least 1% relative abundance in at least one of the analyzed groups.

The overall relative abundance of bacterial genera in the lumen and mucosa along the analyzed intestinal regions is shown in Figure 5. In both luminal and mucosal samples, the most dominating taxa were *Lachnospiraceae NK4A136 group*, *Turicibacter* sp. *and Lachnospiraceae other*, *Escherichia-Shigella *sp. and *Romboutsia *sp. (Figure 5).

## 4. Discussion

Our human study provides for the first time an insight into the developing microbiome of the gastrointestinal tract of four surgically treated preterm infants, considering potential differences of the luminal and mucosal microbiome. Our mouse model analyzed correspondingly confirmed the data situation and gave our findings of differences in the luminal and mucosal microbiome to be considered before performing interventions, an overarching statement. Following the advent of refined microbiological techniques, there are a plethora of studies describing the developing gastrointestinal microbiome of healthy newborns and infants [39]. However, due to accessibility, the majority of these studies are based on a description of the fecal microbiome [40]. Additionally, it is a well-known fact that the bacterial microbiome differs significantly along the sections of the gastrointestinal tract [23]. In 2013, Barrett and coworkers described the microbial diversity and stability of the preterm neonatal ileum and colon of two infants and found that the human infant ileum and colon are dominated by bifidobacteria and the microbiota of the neonatal ileum/colon in ileostomy/colostomy infants is dynamic and unstable, with large changes observed at genus level over the duration of this study [41]. Nevertheless, potential differences of the mucosal and luminal microbiome have not been examined in detail before.

We were able to reveal significantly higher abundances in the mucosa-associated microbiota in two taxa compared to the luminal contents *(Cupriavidus* and *Ralstonia*), and one taxon showed a trend to become significant (*Elizabethkingia)* by DSeq2 analysis over all infants included. *Ralstonia *sp. and *Cupriavidus *sp. are overlapping genera known as environmental Gram-negative bacteria belonging to the class Betaproteobacteria of low virulence, rarely causing infections in nonimmunosuppressed humans [42,43]. Nevertheless, the gastrointestinal tract of neonates suffering from necrotizing enterocolitis was already described in a study by Smith et al. in 2011 to be colonized with *Ralstonia *sp. in seven out of eight studied cases [44]. In our study, *Ralstonia *sp. reads were present in all analyzed mucosa samples, although the relative abundance of the genus ranged from 0.05% to 1.24%, putting the biological or pathogenic relevance into question. Similarly, the occurrence of *Cupriavidus *sp. in all mucosa samples but in only one luminal sample, with relative abundances of 0% up to 1.63%, caused a significant difference between the mucosa and lumen. However, the biological importance also remains to be viewed critically due to the low relative frequency. In our mice samples, the relative abundance of *Cupriavidus *sp. also did not exceed the 1% threshold in any of the analyzed groups. *Elizabethkingia *sp. is a frequent but rarely pathogenic Gram-negative bacterium whose presence in children was reviewed in detail in Dziuban et al. in 2017 [45]. From intestinal studies, we know that the genus *Elizabethkingia *sp. is present in the mucosa of ulcerative colitis (UC) patients and significantly higher in exacerbated UC [43]. In our infant samples, we found an increased abundance of this genus in the mucosa compared to the lumen. From several studies on hospitalized patients, we know the genus was present in the hospital environment, and an uptake during the hospitalization period of the patients cannot be ruled out [44]. The same holds true for the infants included in the present study who all suffered from severe diseases necessitating the creation of an enterostomy. As a potential pathogen, monitoring of the *Elizabethkingia *ssp. load might be of interest, and nutritional interventions might be considered to prevent the colonization of infant’s mucosa with this genus. In the murine study cohort, the genus was present in the mucosa with significantly different abundance compared to the lumen only in the ileum but with a relative abundance of 0.05% (133 total reads), as such questioning the biological relevance of the genus in mice.

The lack of more significantly different taxa in all hierarchical levels might be caused by the low sample number as well as the interindividual differences of the patients. An insufficient washing procedure and residual nucleic acids of luminal taxa at the mucosa samples might be an additional explanation, although this was considered and performed at the best possible. The significant difference in PD whole tree diversity analysis between the mucosa and lumen might be based on biological conditions, but the number of samples analyzed here was limited and thus should be considered as preliminary results. The five most abundant genera that were found in the samples with different frequencies (*Clostridium sensu stricto 1*, *Enterococcus *sp., *Staphylococcus *sp., *Streptococcus *sp. and *Veillonella *sp.) were also found in the same manner in other studies on children’s gut microbiome [12]. For comparative analysis with further studies on children’s samples, we uploaded all data to the ENA archive, and the microbial pattern of not significantly different taxa can be reviewed in the provided Supplementary Material.

In our comparable study in mice, we also report significant differences between mucosa- and lumen-associated microbiota in the intestinal regions (jejunum, ileum and colon). Similarly, we encountered differences for each of the sample materials along the course of the GI tract. Alpha and beta diversity calculations showed statistically significant alterations between the sample sources (luminal vs. mucosal) within an intestinal region as well as within one sample type along the GI tract sections. Taxa with the highest relative abundance significantly different between the mucosa and lumen in the mouse jejunum were *Turicibacter *sp., *Lachnospiraceae NK4A136 *sp., *Escherichia-Shigella *sp. and *Romboutsia *sp. Considered more closely, *Turicibacter* is a genus in the phylum Firmicutes and frequently detected in GI tracts of humans and animals [46]. In our study, we found the genus *Turicibacter* in both sample types but overrepresented in the lumen compared to the mucosa-associated microbiome in all intestinal regions analyzed. This is in contradiction to the results of a recently published study on the comparison of luminal and mucosa-associated microbiota in mice reporting that the genus *Turicibacter* was associated with the mucosa in the duodenum [47]. The relative abundance decreased in our study from the jejunum to the ileum and to the colon, suggesting a main functional role of the genus in the upper GI tract. Although the genus was published to be present in human fecal samples too, no reads mapped to this taxon in infants-derived samples in our study. *Lachnospiraceae NK4A136 *sp. in mice was a very dominant, mucosa-associated genus corresponding to former studies [47], with slightly decreasing abundance from the jejunum to the colon indicating fermentative functions in the upper GI tract as altered in other functional studies [48]. *Romboutsia* significantly differed between the mucosa and lumen in the jejunum and ileum, with the far highest relative abundance was in the lumen of the ileum, confirming the results of former studies on the fermentative action of this genus in the small intestine of rats [49] as well as humans [50], although not present in our data of infants. *Shigella* and *Escherichia* are closely related strains, both belonging to the family *Enterobacteriaceae* and both with possibly pathogenic potential [51]. The occurrence of which should be observed in samples and possibly treatment with beneficial microbes should be considered.

Differences between the analyzed intestinal regions in the mucosa and lumen, e.g., characterized by *Romboutsia*, *Turicibacter*, *Lachnospiraceae NK4A136* or *Enterobacter*, were of course expected due to the different functional requirements of the regions (Supplementary Tables S4 and S5).

Limitations of our study performed in infants are the low number of included patients and its observational design. Nevertheless, the creation of enterostomies is a relatively rarely performed procedure in infants suffering from severe diseases such as necrotizing enterocolitis, meconium-related ileus or spontaneous ileal perforation, and data on these diseases are of immense value. Further, the upload of these data to public databases is an important way to get more data for comparative analysis. In order to confirm our findings and deepen the understanding of the bacterial microbiome in the different sections of the gastrointestinal tract in infants larger multicentric studies are necessary.

In conclusion, we found a different pattern of mucosal and luminal bacterial microbiota in surgically treated infants, although only a small sample cohort on four ileum-derived samples was available. Our corresponding study on the different murine intestinal regions along the GI tract showed differences all over the intestinal region that should be characterized and considered regarding the effect of potential nutritional supplementation as mucosa- and lumen-associated microbiota might be affected in different ways and have different tasks for a healthy microbial community.

## Figures and Tables

**Table 1:** Detailed clinical information of the four infants included in the measurement of potential differences of the luminal and mucosal microbiome.

Patient ID	Gender	Gestational Age (weeks)	Birth Weight (g)	Diagnosis	Age at Creation Stoma	GI Region	Age at Closure Stoma
1	male	27 + 6	720	Meconium-related ileus	5 days	ileostomy	2 months
3	female	25	600	Spontaneous perforation in ileum	4 days	ileostomy	3 months
4	male	29 + 2	650	Meconium-related ileus	3 days	ileostomy	4 months
5	male	24 + 5	720	Necrotizing enterocolitis	27 days	ileostomy	9 months

**Table 2:** Alpha diversity (Shannon, observed species, PD.faith) and beta diversity (weighted UniFrac) calculations of mouse-derived samples. Comparisons were calculated for the tissue differences MU: mucosa, LU: lumen, *: statistically significant with a *p*-value smaller than 0.05.

Jejunum (MU vs. LU)	R2	*p*-Value	adj. *p*-Value
Shannon div. index		0.148	0.445
Observed species		0.016 *	0.047 *
PD.faith		0.844	
Adonis (weighted UniFrac)	0.265	0.015 *	0.045 *
Ileum (MU vs. LU)			
Shannon div. index		0.547	0.742
Observed species		0.055	0.080
PD.faith		0.844	
Adonis (weighted UniFrac)	0.657	0.201	0.302
Colon (MU vs. LU)			
Shannon div. index		0.742	0.742
Observed species		0.195	0.195
PD.faith		0.383	
Adonis (weighted UniFrac)	0.057	0.437	0.437
MUCOSA Shannon		0.046 *	
Jejunum vs. Ileum		0.250	0.330
Jejunum vs. Colon		0.039 *	0.156
Ileum vs. Colon		0.078	0.156
Colon vs. feces		0.640	0.640
MUCOSA observed species		0.745	
MUCOSA Adonis weighted UniFrac	0.366	0.002 *	
Jejunum vs. Ileum	0.350	0.155	0.200
Jejunum vs. Colon	0.400	0.001 *	0.004 *
Ileum vs. Colon	0.103	0.002 *	0.004 *
Colon vs. feces	0.074	0.324	0.324
LUMEN Shannon		0.005 *	
Jejunum vs. Ileum		0.945	0.945
Jejunum vs. Colon		0.023 *	0.047 *
Ileum vs. Colon		0.016 *	0.047 *
Colon vs. feces		0.844	0.945
LUMEN observed species		0.083	
LUMEN Adonis weighted UniFrac	0.495	0.001 *	
Jejunum vs. Ileum	0.075	0.260	0.347
Jejunum vs. Colon	0.578	0.001 *	0.004 *
Ileum vs. Colon	0.486	0.002 *	0.004 *
Colon vs. feces	0.009	0.895	0.895

## Supplementary Materials

The following are available online at https://www.mdpi.com/2072-6643/13/3/1030/s1. Table S1: Human-derived samples: absolute reads distribution and relative abundance at genus level per sample. Table S2: DSeq2 analysis of mucosa- and lumen-derived human samples at the genus level. Table S3: DeSeq genus-level analysis of lumen vs. mucosa-derived sample in jejunum, ileum and colon regions. Table S4: DeSeq analysis at genus level in mice in the mucosa between jejunum, ileum and colon regions. Table S5: DeSeq analysis at genus level in mice in the lumen between jejunum, ileum and colon regions. Table S6: Genus level analysis of mouse sample: absolute read numbers and relative abundances per group.

## References

[1] Human Microbiome Project Consortium (2012). A Framework for Human Microbiome Research. Nature.

[2] Dekaboruah E., Suryavanshi M.V., Chettri D., Verma A.K. (2020). Human Microbiome: An Academic Update on Human Body Site Specific Surveillance and its Possible Role. Arch. Microbiol..

[3] Milani C., Duranti S., Bottacini F., Casey E., Turroni F., Mahony J., Belzer C., Delgado Palacio S., Arboleya Montes S., Mancabelli L. (2017). The First Microbial Colonizers of the Human Gut: Composition, Activities, and Health Implications of the Infant Gut Microbiota. Microbiol. Mol. Biol. Rev..

[4] Yang I., Corwin E.J., Brennan P.A., Jordan S., Murphy J.R., Dunlop A. (2016). The Infant Microbiome: Implications for Infant Health and Neurocognitive Development. Nurs. Res..

[5] Willis A.D., Minot S.S. (2020). Strategies to Facilitate Translational Advances from Microbiome Surveys. Trends Microbiol..

[6] Diaz Heijtz R. (2016). Fetal, Neonatal, and Infant Microbiome: Perturbations and Subsequent Effects on Brain Development and Behavior. Semin. Fetal. Neonatal Med..

[7] Miqdady M., Al Mistarihi J., Azaz A., Rawat D. (2020). Prebiotics in the Infant Microbiome: The Past, Present, and Future. Pediatr. Gastroenterol. Hepatol. Nutr..

[8] Selma-Royo M., Tarrazo M., Garcia-Mantrana I., Gomez-Gallego C., Salminen S., Collado M.C. (2019). Shaping Microbiota during the First 1000 Days of Life. Adv. Exp. Med. Biol..

[9] Plaza-Diaz J., Ruiz-Ojeda F.J., Gil-Campos M., Gil A. (2018). Immune-Mediated Mechanisms of Action of Probiotics and Synbiotics in Treating Pediatric Intestinal Diseases. Nutrients.

[10] Fassarella M., Blaak E.E., Penders J., Nauta A., Smidt H., Zoetendal E.G. (2021). Gut Microbiome Stability and Resilience: Elucidating the Response to Perturbations in Order to Modulate Gut Health. Gut.

[11] Hill C.J., Lynch D.B., Murphy K., Ulaszewska M., Jeffery I.B., O’Shea C.A., Watkins C., Dempsey E., Mattivi F., Tuohy K. (2017). Evolution of Gut Microbiota Composition from Birth to 24 Weeks in the INFANTMET Cohort. Microbiome.

[12] Gritz E.C., Bhandari V. (2015). The Human Neonatal Gut Microbiome: A Brief Review. Front. Pediatr..

[13] Kim G., Bae J., Kim M.J., Kwon H., Park G., Kim S.J., Choe Y.H., Kim J., Park S.H., Choe B.H. (2020). Delayed Establishment of Gut Microbiota in Infants Delivered by Cesarean Section. Front. Microbiol..

[14] Van den Elsen L.W.J., Garssen J., Burcelin R., Verhasselt V. (2019). Shaping the Gut Microbiota by Breastfeeding: The Gateway to Allergy Prevention?. Front. Pediatr..

[15] O’Neill I.J., Sanchez Gallardo R., Saldova R., Murphy E.F., Cotter P.D., McAuliffe F.M., van Sinderen D. (2020). Maternal and Infant Factors that Shape Neonatal Gut Colonization by Bacteria. Expert Rev. Gastroenterol. Hepatol..

[16] Peterson J.A., Patton S., Hamosh M. (1998). Glycoproteins of the Human Milk Fat Globule in the Protection of the Breast-Fed Infant against Infections. Biol. Neonate.

[17] Shang J., Piskarev V.E., Xia M., Huang P., Jiang X., Likhosherstov L.M., Novikova O.S., Newburg D.S., Ratner D.M. (2013). Identifying Human Milk Glycans that Inhibit Norovirus Binding using Surface Plasmon Resonance. Glycobiology.

[18] Backhed F., Roswall J., Peng Y., Feng Q., Jia H., Kovatcheva-Datchary P., Li Y., Xia Y., Xie H., Zhong H. (2015). Dynamics and Stabilization of the Human Gut Microbiome during the First Year of Life. Cell. Host Microbe.

[19] Ximenez C., Torres J. (2017). Development of Microbiota in Infants and its Role in Maturation of Gut Mucosa and Immune System. Arch. Med. Res..

[20] Tanaka M., Nakayama J. (2017). Development of the Gut Microbiota in Infancy and its Impact on Health in Later Life. Allergol. Int..

[21] Thiemann S., Smit N., Roy U., Lesker T.R., Galvez E.J.C., Helmecke J., Basic M., Bleich A., Goodman A.L., Kalinke U. (2017). Enhancement of IFNgamma Production by Distinct Commensals Ameliorates Salmonella-Induced Disease. Cell. Host Microbe.

[22] Vuik F., Dicksved J., Lam S.Y., Fuhler G.M., van der Laan L., van de Winkel A., Konstantinov S.R., Spaander M., Peppelenbosch M.P., Engstrand L. (2019). Composition of the Mucosa-Associated Microbiota along the Entire Gastrointestinal Tract of Human Individuals. United. Eur. Gastroenterol. J..

[23] Bashir M., Prietl B., Tauschmann M., Mautner S.I., Kump P.K., Treiber G., Wurm P., Gorkiewicz G., Hogenauer C., Pieber T.R. (2016). Effects of High Doses of Vitamin D3 on Mucosa-Associated Gut Microbiome Vary between Regions of the Human Gastrointestinal Tract. Eur. J. Nutr..

[24] Liu H., Zeng X., Zhang G., Hou C., Li N., Yu H., Shang L., Zhang X., Trevisi P., Yang F. (2019). Maternal Milk and Fecal Microbes Guide the Spatiotemporal Development of Mucosa-Associated Microbiota and Barrier Function in the Porcine Neonatal Gut. BMC Biol..

[25] Kurath-Koller S., Neumann C., Moissl-Eichinger C., Kraschl R., Kanduth C., Hopfer B., Pausan M.R., Urlesberger B., Resch B. (2020). Hospital Regimens Including Probiotics Guide the Individual Development of the Gut Microbiome of very Low Birth Weight Infants in the First Two Weeks of Life. Nutrients.

[26] Combellick J.L., Shin H., Shin D., Cai Y., Hagan H., Lacher C., Lin D.L., McCauley K., Lynch S.V., Dominguez-Bello M.G. (2018). Differences in the Fecal Microbiota of Neonates Born at Home or in the Hospital. Sci. Rep..

[27] Lundgren S.N., Madan J.C., Emond J.A., Morrison H.G., Christensen B.C., Karagas M.R., Hoen A.G. (2018). Maternal Diet during Pregnancy is Related with the Infant Stool Microbiome in a Delivery Mode-Dependent Manner. Microbiome.

[28] Senn V., Bassler D., Choudhury R., Scholkmann F., Righini-Grunder F., Vuille-Dit-Bile R.N., Restin T. (2020). Microbial Colonization from the Fetus to Early Childhood-A Comprehensive Review. Front. Cell. Infect. Microbiol..

[29] Hodzic Z., Bolock A.M., Good M. (2017). The Role of Mucosal Immunity in the Pathogenesis of Necrotizing Enterocolitis. Front. Pediatr..

[30] Klymiuk I., Bilgilier C., Stadlmann A., Thannesberger J., Kastner M.T., Hogenauer C., Puspok A., Biowski-Frotz S., Schrutka-Kolbl C., Thallinger G.G. (2017). The Human Gastric Microbiome is Predicated upon Infection with Helicobacter Pylori. Front. Microbiol..

[31] McKenna P., Hoffmann C., Minkah N., Aye P.P., Lackner A., Liu Z., Lozupone C.A., Hamady M., Knight R., Bushman F.D. (2008). The Macaque Gut Microbiome in Health, Lentiviral Infection, and Chronic Enterocolitis. PLoS Pathog..

[32] Callahan B.J., McMurdie P.J., Rosen M.J., Han A.W., Johnson A.J., Holmes S.P. (2016). DADA2: High-Resolution Sample Inference from Illumina Amplicon Data. Nat. Methods.

[33] Bolyen E., Rideout J.R., Dillon M.R., Bokulich N.A., Abnet C.C., Al-Ghalith G.A., Alexander H., Alm E.J., Arumugam M., Asnicar F. (2019). Author Correction: Reproducible, Interactive, Scalable and Extensible Microbiome Data Science using QIIME. Nat. Biotechnol..

[34] Afgan E., Baker D., Batut B., van den Beek M., Bouvier D., Cech M., Chilton J., Clements D., Coraor N., Gruning B.A. (2018). The Galaxy Platform for Accessible, Reproducible and Collaborative Biomedical Analyses: 2018 Update. Nucleic Acids Res..

[35] Quast C., Pruesse E., Yilmaz P., Gerken J., Schweer T., Yarza P., Peplies J., Glockner F.O. (2013). The SILVA Ribosomal RNA Gene Database Project: Improved Data Processing and Web-Based Tools. Nucleic Acids Res..

[36] Price M.N., Dehal P.S., Arkin A.P. (2010). FastTree 2—Approximately Maximum-Likelihood Trees for Large Alignments. PLoS ONE.

[37] Chen L., Reeve J., Zhang L., Huang S., Wang X., Chen J. (2018). GMPR: A Robust Normalization Method for Zero-Inflated Count Data with Application to Microbiome Sequencing Data. PeerJ.

[38] Love M.I., Huber W., Anders S. (2014). Moderated Estimation of Fold Change and Dispersion for RNA-Seq Data with DESeq2. Genome Biol..

[39] Nguyen T.T.B., Chung H.J., Kim H.J., Hong S.T. (2019). Establishment of an Ideal Gut Microbiota to Boost Healthy Growth of Neonates. Crit. Rev. Microbiol..

[40] Robertson R.C., Manges A.R., Finlay B.B., Prendergast A.J. (2019). The Human Microbiome and Child Growth-First 1000 Days and Beyond. Trends Microbiol..

[41] Barrett E., Guinane C.M., Ryan C.A., Dempsey E.M., Murphy B.P., O’Toole P.W., Fitzgerald G.F., Cotter P.D., Ross R.P., Stanton C. (2013). Microbiota Diversity and Stability of the Preterm Neonatal Ileum and Colon of Two Infants. Microbiologyopen.

[42] Zhang Z., Deng W., Wang S., Xu L., Yan L., Liao P. (2017). First Case Report of Infection Caused by Cupriavidus Gilardii in a Non-Immunocompromised Chinese Patient. IDCases.

[43] Yahya R., Alyousef W., Omara A., Alamoudi S., Alshami A., Abdalhamid B. (2017). First Case of Pneumonia Caused by Cupriavidus Pauculus in an Infant in the Gulf Cooperation Council. J. Infect. Dev. Ctries.

[44] Smith B., Bode S., Petersen B.L., Jensen T.K., Pipper C., Kloppenborg J., Boye M., Krogfelt K.A., Molbak L. (2011). Community Analysis of Bacteria Colonizing Intestinal Tissue of Neonates with Necrotizing Enterocolitis. BMC Microbiol..

[45] Dziuban E.J., Franks J.L., So M., Peacock G., Blaney D.D. (2018). Elizabethkingia in Children: A Comprehensive Review of Symptomatic Cases Reported from 1944 to 2017. Clin. Infect. Dis..

[46] Cuiv P.O., Klaassens E.S., Durkin A.S., Harkins D.M., Foster L., McCorrison J., Torralba M., Nelson K.E., Morrison M. (2011). Draft Genome Sequence of Turicibacter Sanguinis PC909, Isolated from Human Feces. J. Bacteriol..

[47] Wu M., Li P., Li J., An Y., Wang M., Zhong G. (2020). The Differences between Luminal Microbiota and Mucosal Microbiota in Mice. J. Microbiol. Biotechnol..

[48] Safari Z., Bruneau A., Monnoye M., Mariadassou M., Philippe C., Zatloukal K., Gerard P. (2020). Murine Genetic Background Overcomes Gut Microbiota Changes to Explain Metabolic Response to High-Fat Diet. Nutrients.

[49] Gerritsen J., Hornung B., Renckens B., van Hijum S.A.F.T., Martins Dos Santos V.A.P., Rijkers G.T., Schaap P.J., de Vos W.M., Smidt H. (2017). Genomic and Functional Analysis of Romboutsia Ilealis CRIB(T) Reveals Adaptation to the Small Intestine. PeerJ.

[50] Gerritsen J., Umanets A., Staneva I., Hornung B., Ritari J., Paulin L., Rijkers G.T., de Vos W.M., Smidt H. (2018). Romboutsia Hominis Sp. Nov., the First Human Gut-Derived Representative of the Genus Romboutsia, Isolated from Ileostoma Effluent. Int. J. Syst. Evol. Microbiol..

[51] Van den Beld M.J.C., Warmelink E., Friedrich A.W., Reubsaet F.A.G., Schipper M., de Boer R.F., Notermans D.W., Petrignani M.W.F., van Zanten E., Rossen J.W.A. (2019). Incidence, Clinical Implications and Impact on Public Health of Infections with Shigella Spp. and Entero-Invasive Escherichia Coli (EIEC): Results of a Multicenter Cross-Sectional Study in the Netherlands during 2016–2017. BMC Infect. Dis..

